# Water–use efficiency of dryland wheat in response to mulching and tillage practices on the Loess Plateau

**DOI:** 10.1038/srep12225

**Published:** 2015-07-20

**Authors:** Li-fang Wang, Zhou-ping Shangguan

**Affiliations:** 1 State Key Laboratory of Soil Erosion and Dryland Farming on the Loess Plateau, Northwest A&F University, Yangling, Shaanxi 712100, China; 2 Institute of Soil and Water Conservation, Chinese Academy of Sciences and Ministry of Water Resources, Yangling, Shaanxi 712100, China

## Abstract

Mulching and tillage are widely considered to be major practices for improving soil and water conservation where water is scarce. This paper studied the effects of FM (flat mulching), RFM (ridge-furrow mulching), SM (straw mulching), MTMC (mulching with two materials combined), MOM (mulching with other materials), NT (no-tillage) ST (subsoiling tillage) and RT (rotational tillage) on wheat yield based on a synthesis of 85 recent publications (including 2795 observations at 24 sites) in the Loess Plateau, China. This synthesis suggests that wheat yield was in the range of 259–7898 kg ha^−1^ for FM and RFM. The sequence of water use efficiency (WUE) effect sizes was similar to that of wheat yield for the practices. Wheat yields were more sensitive to soil water at planting covered by plastic film, wheat straw, liquid film, water-permeable plastic film and sand compared to NT, ST and RT. RFM and RT increased the yields of wheat by 18 and 15%, respectively, and corresponding for WUE by 20.11 and 12.50%. This synthesis demonstrates that RFM was better for avoiding the risk of reduced production due to lack of precipitation; however, under conditions of better soil moisture, RT and MTMC were also economic.

Water shortage is the major constraint to agricultural production in rainfed farming systems of arid and semi-arid areas. A large part of rainfed farming systems in China is located in the semi-arid Loess Plateau^1^. Most of the farmland on the Loess Plateau relies solely on the rare and erratic rainfall; and intensive evaporation and low rainwater use efficiency always constrain agricultural production and sustainable development^2^. In light of the expanding human population, combined with changing lifestyles and water scarcity, finding optimal ways to conserve limited water resources and improve crop water use efficiency (WUE) by quantifying current conservation tillage practices is becoming a key issue in sustainable crop productivity.

Winter wheat yield and WUE in dryland areas depend strongly on soil-available water. Studies by Shangguan *et al.*^3^ in the northern Loess Plateau of China and by Musick *et al.*^4^ in the southern Great Plains (SGP) of the U.S. have demonstrated the importance of storing soil water during fallow periods for increasing wheat yield and WUE, and the relationship between soil water storage at planting and yield emphasizes the importance of practices that capture both fallow and in-season rainfall^5^. The selection of optimal management practices in different regions has received increasing attention from academics; for example, Liao *et al.*^6^ reported that controlled traffic treatments conserve the natural soil structure by zero tillage and straw cover, thereby reducing evaporation. In addition to promoting water and soil conservation, proper field management practices provide soil temperature moderation, soil structure improvement, soil nutrient effects, and the control of soil salinity, crop quality, and weeds^7^. A combination of these benefits is thus highly important for achieving adequate food production to meet the needs of the ever-increasing human population.

Conservation tillage systems have been demonstrated in the semiarid region of the Loess Plateau, increasing WUE by 11%^8^. No-tillage (NT) and subsoiling tillage (ST) practices, as well as NT combined with ST, show promise in increasing soil water storage, improving WUE and increasing crop yield^9^. Soil compaction can adversely affect root growth, the uptake of water and nutrients, and crop yield^10^. These adverse effects can be reduced by ST, which aims to break the compacted layers underneath the plow layer and increase the porosity of the A horizon. ST is a key component of conservation tillage, as the practice improves soil structure, reduces soil strength, eliminates soil compaction, and increases yield and WUE^11^. Conservation tillage uses either minimum-tillage or NT practices to maximize straw coverage, which can increase soil water content and improve soil structure, thereby increasing yield and WUE and advancing the sustainable development of agriculture worldwide^12^,^13^,^14^. However, although several authors have analyzed the effects of management practices in determining yield and WUE, a consensus on the relative significance of these practices in different regions has yet to be achieved, indicating a need for further study on the effects of conservation practices on grain yield and WUE in particular areas.

The Loess Plateau of China is located along the middle reaches of the Yellow River. The region has a total area of over 642,000 km^2^, a population of 82 million, and a semiarid climate with an average annual precipitation of 300–600 mm^15^,^16^. Researchers face a serious challenge in the relatively small number of actual observations used to estimate the large-scale yield and WUE changes due to different mulching and tillage practices, and little is known about the long-term effects of these practices on water conservation, crop yield, evapotranspiration (ET), and WUE in the semiarid Loess Plateau of northern China. Consequently, a more accurate method is required to estimate the effect sizes of management practices on crop production. The present meta-analysis sheds light on the contributions of these practices, clarifies their impacts, and provides a new, more robust theoretical model.

This meta-analysis has two objectives: (1) to quantify the potential effect sizes of the mulching practices of flat mulching (FM), ridge-furrow mulching (RFM), straw mulching (SM), mulching with two materials combined (MTMC), and mulching with other materials (MOM), as well as those of the tillage practices of NT, ST, and rotational tillage (RT), on grain yield, WUE, and ET across the Loess Plateau; and (2) to establish relationships between soil water at planting and yield with crop yield, WUE, and ET, as well as to determine the optimum mulching and tillage practices in northwest China. To achieve these objectives, we synthesized the findings of 85 recent publications from the literature in which yield, WUE, and ET were related to conservation management.

## Results

### Yield, WUE, and ET under different mulching and tillage practices

**Figure 1:**
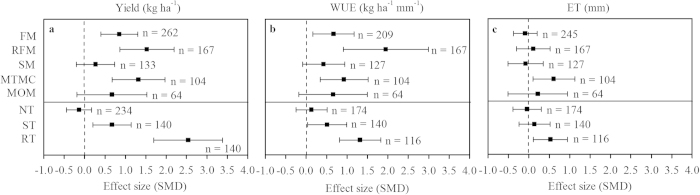
The relative effect sizes of wheat yields, water-use efficiency (WUE), and evapotranspiration (ET) for different mulching and tillage practices on the Loess Plateau. Error bars represent 95% CI, and the values close to the bars represent the corresponding number of observations. FM: flat mulching; RFM: ridge-furrow mulching; SM: straw mulching; MTMC: mulching with two materials combined; MOM: mulching with other materials; NT: no-tillage; ST: subsoiling tillage; RT: rotational tillage.

The meta-analysis results showed different responses of wheat yields to mulching and tillage practices (Fig. 1a, Table 1). The mean yield effect sizes of FM, RFM, MTMC, ST, and RT were 0.85 (0.40–1.29), 1.53 (0.86–2.19), 1.32 (0.67–1.97), 0.67 (0.20–1.14), and 2.54 (1.70–3.38), respectively, and their 95% CIs did not include zero, showing a significant positive effect of conservation tillage on wheat yields. The yields were also increased by SM (0.27, –0.20 to 0.74) and MOM (0.67, –0.19 to 1.53) and, conversely, the tillage practice of NT had a negative effect; however, neither were significant (Fig. 1a). The yield effect sizes of the different mulching and tillage practices were ranked in the order of RFM > MTMC > FM > MOM > SM and RT > ST > NT, respectively (Fig. 1a). Grain yield was in the range of 259–7898 and 869–6909 kg ha^−1^ under the different mulching and tillage practices, respectively. On average, the mulching practices of RFM and MTMC increased yield relative to CT by 18 and 28%, respectively; and the tillage practice of RT increased yield relative to CT by 15% (Table 2). Both the yield and yield responses to mulching and tillage practices relative to CT had a wide range of variation.

WUE was generally increased by different mulching and tillage practices (Fig. 1b, Table 1). The effect sizes of FM (0.67, 0.17–1.17), RFM (1.95, 0.91–2.99), MTMC (0.93, 0.34–1.52), ST (0.51, 0.03–0.98) and RT (1.32, 0.81–1.83) were significant for increasing WUE, and those of SM (0.43, –0.09 to 0.96), MOM (0.66, –0.18 to 1.49) and NT (0.13, –0.25 to 0.51) were not significant. The RFM method caused the greatest improvement in WUE (20.11%) among mulching practices, following by MTMC (19.71%); RT caused the highest increase in WUE (12.50%) among tillage practices; the sequence of WUE effect sizes was similar to that of wheat yield for the different mulching and tillage practices. (Fig. 1b). Crop WUE was in the range of 0.6–26.8 and 3.5–23.0 kg ha^−1^mm^−1^ for the different mulching and tillage practices, respectively (Table 2).

The mean effect sizes of ET were shown in Fig. 1c. For the response of mulching and tillage practices to ET, FM (–0.09, –0.38 to 0.21), RFM (0.11, –0.30 to 0.53), SM (–0.08, –0.51 to 0.35), MOM (0.22, –0.51 to 0.96), NT (–0.04, –0.39 to 0.32) and ST (0.14, –0.24 to 0.53) showed no significant change; while MTMC (0.61, 0.10–1.11) and RT (0.53, 0.11–0.96) resulted in significant increase (Fig. 1c). Seasonal ET was in the range of 112–555 and 123–523 mm under the different mulching and tillage practices, respectively, but the means for 259–355 mm for the different practices showed a relatively narrow range.

### Relationship between grain yield and soil water at planting

**Figure 2:**
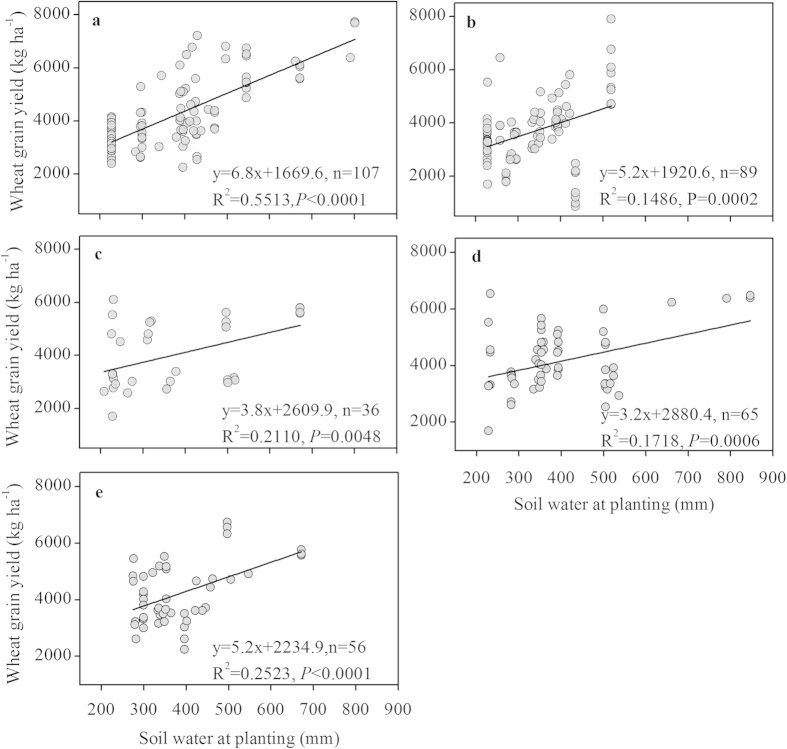
Relationships between grain yield of wheat and soil water content at sowing under five different mulching practices (including conventional tillage) based on published data on the Loess Plateau. **a**: flat mulching (FM); **b**: ridge-furrow mulching (RFM); **c**: straw mulching (SM); **d**: mulching with two materials combined (MTMC); **e**: mulching with other materials (MOM).

**Figure 3:**
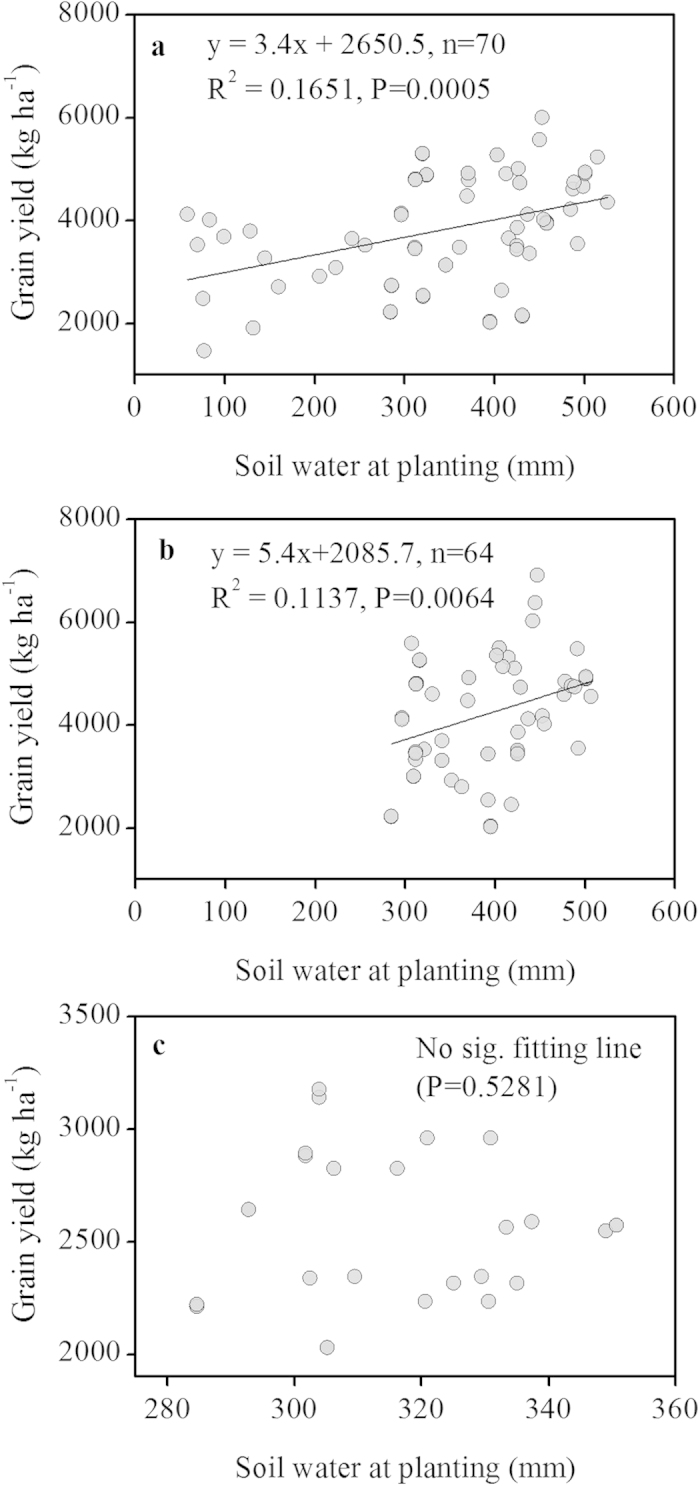
Relationship between grain yield of wheat and soil water content at sowing under three different tillage practices (including conventional tillage) based on published data on the Loess Plateau. **a**: no-tillage (NT); **b**: subsoiling tillage (ST); **c**: rotational tillage (RT).

The yields increased linearly with the soil water at planting. The results showed that the relationship between grain yield and soil water at planting under different management practices of FM, RFM, SM, MTMC, MOM, NT and ST were significant (*P *< 0.01) (Fig. 2, Fig. 3a,b). The relationship between the grain yield and soil water at planting was not significant under the tillage practice of RT (*P *> 0.05) (Fig. 3c). The degrees of correlation between soil water at planting and wheat yield under different mulching and tillage practices were ranked in the orders of FM > MOM > SM > MTMC > RFM and NT > ST > RT, respectively.

### Water consumption characteristics

**Figure 4:**
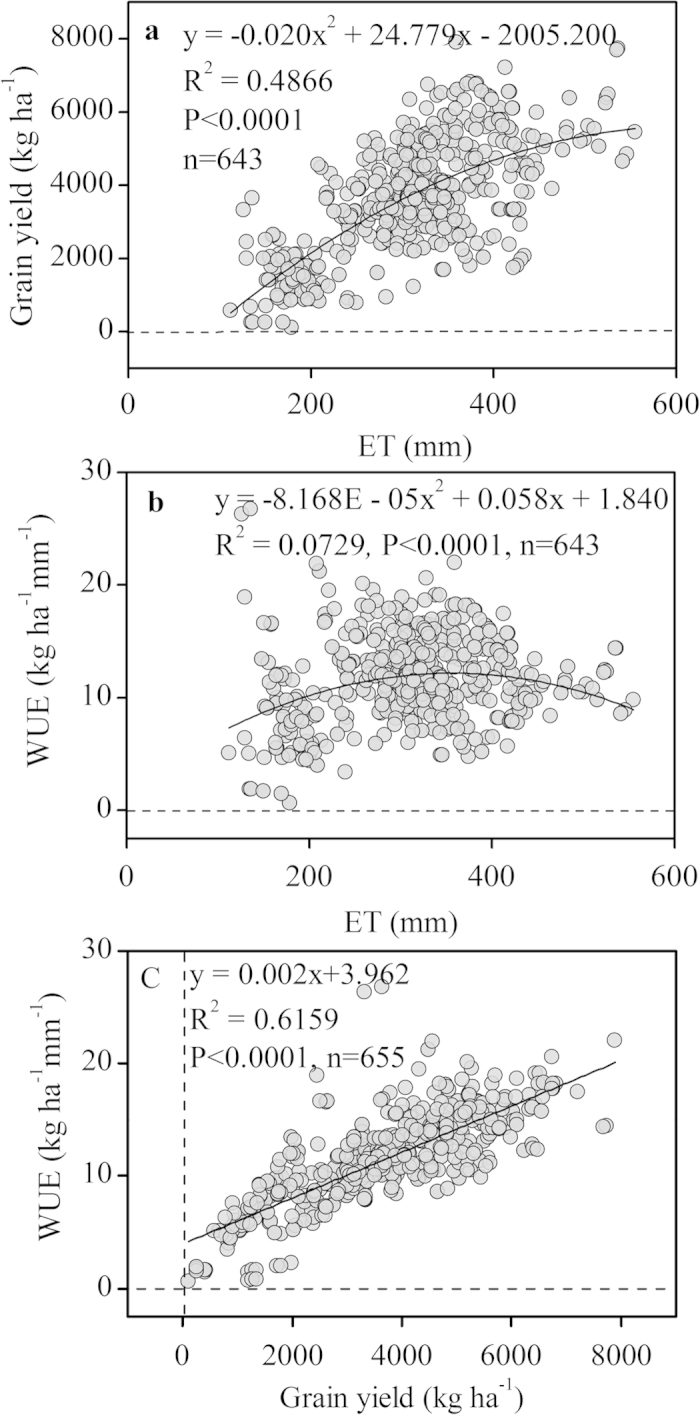
Relationships of yield, water-use efficiency (WUE), and evapotranspiration (ET) of wheat under five different mulching practices on the Loess Plateau. **a**: relationship between ET and grain yield; **b**: relationship between ET and WUE; **c**: relationship between grain yield and WUE. The relationships are shown by regression equations and regression lines.

**Figure 5:**
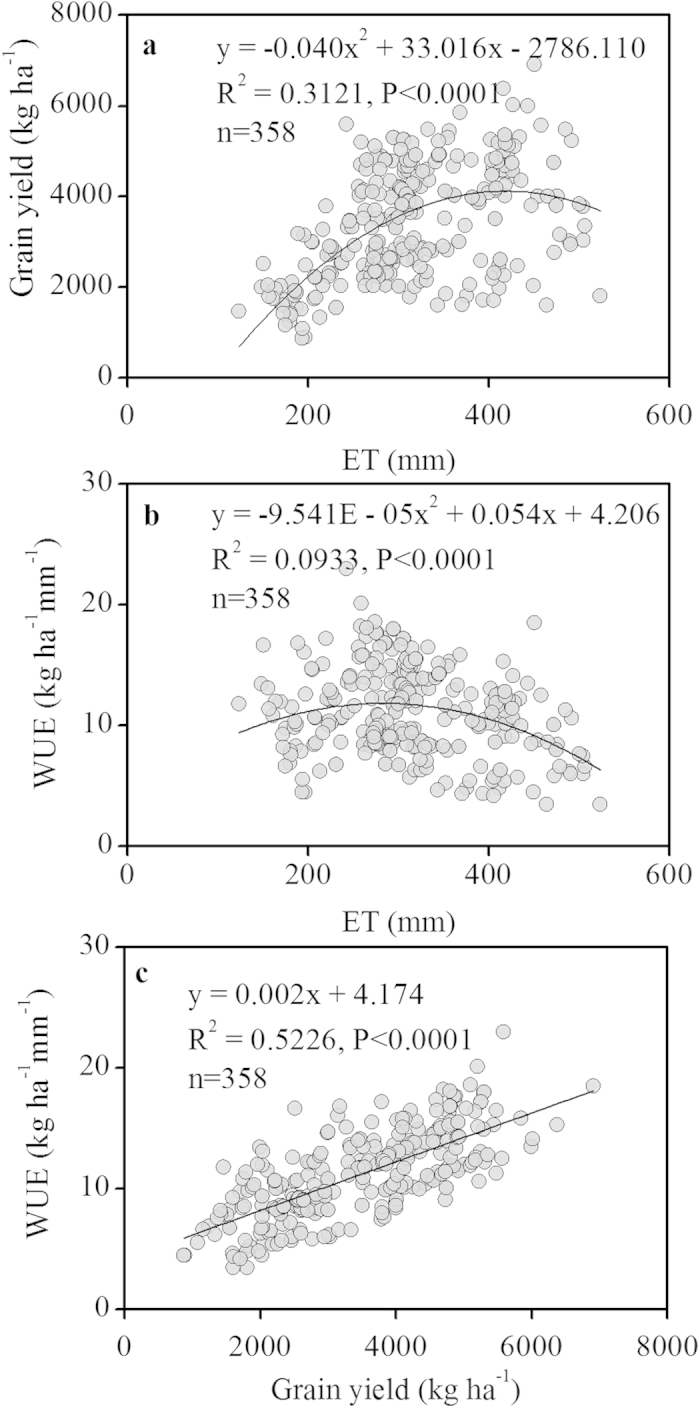
Relationships of yield, water-use efficiency (WUE), and evapotranspiration (ET) of wheat under three different tillage practices on the Loess Plateau. **a**: relationship between ET and grain yield; **b**: relationship between ET and WUE; **c**: relationship between grain yield and WUE. The relationships are shown by the regression equations and regression lines.

In the Loess Plateau, the relationships between grain yield and seasonal ET under different mulching and tillage practices were best described by two quadratic functions obtained by regression analysis after pooling all data sets (Y = −0.020x^2 ^+ 24.779x − 2005.200, Fig. 4a; Y = −0.040×^2^ + 33.016x − 2786.110, Fig. 5a). Grain yield decreased when ET exceeded a certain critical value, approximately 619 mm under the different mulching practices and 413 mm under the different tillage practices in the present study. Wheat required a minimum ET of 112 mm for any grain yield under the five mulching practices (Fig. 4a). Regression analysis also produced parabolic relationships between WUE and ET under different mulching and tillage practices (*P *< 0.0001). WUE reached a maximum at an ET of 355 mm under the different mulching practices and 283 mm under the different tillage practices, and then greatly decreased (Figs 4b and 5b). The relationship between WUE and yield was shown in Fig. 4c and Fig. 5c. WUE increased linearly as yield increased in the Loess Plateau. In this region, a yield increase of 1 kg ha^−1^ led to an increase in WUE of 0.002 kg ha^−1^ mm^−1^ under the different mulching and tillage practices (R^2^ = 0.6159, *P *< 0.0001 and R^2^ = 0.5226, *P *< 0.0001, respectively). The results indicate that a higher WUE is generally achieved under higher yields.

## Discussion

### Relationships of grain yield and soil water at planting under different mulching and tillage practices

The variations of yield and WUE may be attributable to the amount of rainfall during the crop growing seasons and the soil water available at planting. Musick *et al.*^4^ showed that wheat yield was linearly related to the soil water stored at planting, and this positive relationship was stronger than that of yield with seasonal water use (250 mm) in the semi-arid U.S. southern High Plains. In the Loess Plateau, grain yields increased linearly with the soil water available at planting, and this relationship was significant under all mulching and tillage practices (*P *< 0.05) except for RT (Fig. 2, Fig. 3). The available soil water at wheat planting time depended on the tillage system used in the summer fallow^17^; however, interestingly, wheat yields under management practices of RFM and RT were less dependent on soil water at planting, and wheat yields were more sensitive to soil water at planting for mulching than for tillage practices.

### Relationships of grain yield, WUE, and ET under different mulching and tillage practices

The wheat yield–ET relationship has been previously reported in different regions and under different crop management practices. Stone and Schlegel^18^ summarized dryland wheat data for 1974–2004 and found a linear relationship between wheat yield and ET with perennial water-short in the USA’s west-central Great Plains; Huang *et al.*^19^ and Li *et al.*^12^ estimated similar results with growing season precipitation of 181 mm on the Loess Plateau. The relationships between grain yields and seasonal ET under the different mulching and tillage practices in this study were best described by two quadratic functions (Figs 4a and 5a) when pooling data sets from the Loess Plateau. Our results agreed with previously estimated results for the Loess Plateau under long-term nitrogen fertilization regimes^20^. Wheat required a minimum ET of 112 mm to produce yield under the five mulching practices (Fig. 4a); this value was higher than the 84 mm previously reported for wheat in the North China Plain^21^, as well as lower than the 206 mm for dryland and irrigated wheat reported by Musick *et al.*^4^ in the southern plains of the U.S. and the 156 mm reported in the Mediterranean region^22^. These differences may be due to the relatively small size of the Loess Plateau; the area experiences almost uniformly low precipitation, and yield is therefore more reliant on precipitation, soil water storage, and crop management. The analysis of the yield-ET relationship provides an important tool for identifying the potential wheat yield and the yield gap between actual and attainable yields under water-limited conditions.

In the Loess Plateau, the variability in the amount and distribution of seasonal precipitation may be a major source of variation in ET and WUE^4^. Significant parabolic relationships between WUE and ET on the Loess Plateau were observed under the different mulching and tillage practices in this study (Figs 4b and 5b). However, Lal and Stewart^23^ indicated that no clear relationship existed between wheat WUE and seasonal ET on the Loess Plateau. Conversely, a positive relationship between WUE and ET was observed in the SGP of the U.S. from pooled dryland and irrigated data. The lack of irrigation on the Loess Plateau may contribute to part of the variation in WUE and ET, and fertilization may significantly contribute to the variation in WUE given the same level of ET^24^.

This study found that WUE increased linearly as yield increased on the Loess Plateau under the different mulching and tillage practices (Figs 4c and 5c); this trend was similar with the results of Lal and Stewart^23^ and Zhong * et al.*^20^ for the same region. However, the WUE-yield relationship was quadratic when the full range of yield was considered^23^,^25^. Our results indicated that higher WUE can generally be achieved with higher yield. Yield increased linearly with WUE; however, the maximum WUE did not correspond to the maximum grain yield in the study. A higher WUE indicates that the crop can obtain a higher yield using less water. Increasing WUE is thus an important method for obtaining a balance between higher wheat yields and lower water supplies in arid and semiarid regions.

### Implications for mulching and tillage practices

The average WUE under the different mulching and tillage practices in the present study was lower than the 14.8 kg ha^−1^mm^−1^ reported by Zhu *et al.*^26^ in a subhumid region of northern China but higher than the 9.0 kg ha^−1^mm^−1^ previously reported by Li *et al.* 27 for the Loess Plateau (both places without irrigation), the present data were within this range. Differences in environmental conditions and management practices among regions may be responsible for the observed yield gaps. Therefore, mulching and tillage practices have great promise for increasing yield because environmental conditions are hard to control.

In semiarid environments with rainfall above the minimum threshold to be benefitted by water storage, water retained in the topsoil is more easily lost by evaporation. The mean effect sizes of yield and WUE under the different mulching practices in this study were significant except for those of SM and MOM, and the effect size of RFM was the largest (Fig. 1a,b). Although SM generally increases yields by enhancing soil water storage^28^, in poorly drained soils or in temperate climates with suboptimal springtime temperatures, residue retention can reduce yields below optimal levels due to decreases in the soil temperature compared to CT, as has been observed in northeastern China^29^. RFM clearly increased wheat yield and WUE, but ET was not increased significantly, in contrast to the effect sizes of other treatments in our study (Fig. 1). Of the mulching practices, RFM may be a sustainable mean of increasing wheat yield and WUE in this semi-arid region.

Winter wheat yields were also significantly affected by the tillage methods in this study. NT, controlled traffic farming is a promising solution for increasing soil water storage, crop yield and WUE compared to CT on the Loess Plateau of China^30^. The mean effect sizes of yield and WUE under the tillage practices of ST and RT in this study were significant (Fig. 1a,b); these results are consistent with those of Zheng *et al.*^31^. The significant contributions of the tillage practices can be explained by the improvement of soil physical properties and greater soil moisture. Many previous studies have indicated that NT may either reduce or increase yields compared to those of CT in drier^32^,^33^. In contrast with the SGP of the U.S., NT is generally unpopular in the Loess Plateau^34^. The effectiveness of conservation tillage on improving WUE and grain yield depends on soil type, crop requirements, rainfall probability, environmental conditions and soil water-storage capacity which vary year-to-year^35^,^36^. He *et al.*^9^ showed that the integration of maize NT seeding and ST into a single-pass operation used less power, fuel, and time in the field, as well as produced higher yield, than a pattern of 4 years of NT coverage plus 1 year of ST. Thus, before conservation tillage practices are widely adopted in any particular region, the suitability of the system must be locally assessed. The positive effect sizes of RT for yield, WUE and ET were the largest among the different tillage practices in this study (Fig. 1a,b). This new tillage mode could solve the soil water loss and low WUE issues that arise from soil compaction under NT, thus improving grain yield and WUE. Increasing the water demand appropriately during growing season might significantly improve the wheat yield and WUE because RT was less dependent on the soil water at planting (Fig. 1, Fig. 3). Therefore, encouraging farmers to apply this tillage practice would contribute to higher yield on the Loess Plateau.

### Uncertainty in the meta-analysis and future perspectives

This synthesis offers the most accurate estimates of potential yield and WUE under different mulching and tillage practices across the Loess Plateau. The strict accuracy of the study is limited, however, due to the uneven distribution of data collected across the region. Some uncertainties are derived from the temporal pattern of grain yield accumulation; many of the studies conducted no long-term observations, which may have added to the uncertainty. Additionally, we ignored the effects of fertilization and soil conditions before sowing. In the future, we hope to focus on the fertilization and soil effects at the sites and the changes in grain yield to determine the functional relationship between these factors and grain yield.

Some progress has been made in recent decades in improving wheat yield and WUE in China’s Loess Plateau, but developing better management strategies remains a challenge for agricultural scientists. The future improvement of yield and WUE may depend on the integration of various management practices, such as mulching, tillage, and fertilization, as well as the adoption of more diversified cropping systems. Such practices will not only result in more sustainable crop productivity but will also provide numerous environmental benefits.

## Method

### Data search and collection

**Figure 6:**
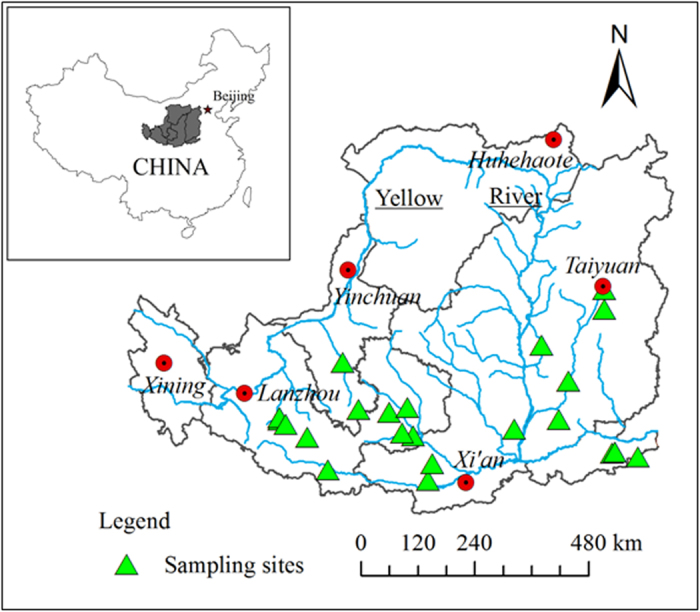
Map of the Loess Plateau, with a map of China enclosed in the upper left. The locations of the wheat field experiments from the peer-reviewed literature used in the meta-analysis are shown on the map. The software ArcGis 9.3 (ESRI, Redlands, California) was used to create the maps.

The relevant literature (1995–2014) on the changes of wheat grain yields, WUE, and ET following the implementation of mulching and tillage practices in the Loess Plateau was searched using the online databases of the Chinese Academy of Sciences (http://www.isiknowledge.com/ and http://www.cnki.net/). After the query results were carefully checked, 2795 observations from 85 studies conducted at 24 sites (Fig. 6, Appendix dataset S1 ) that fitted our selection criteria for the meta-analysis were selected. When the data in question were expressed in the forms of figures or charts, they were transformed into numerical values from their digital versions using Get–Data Graph Digitizer (ver. 2.24, Russian Federation).

To avoid printing-caused distortions, the data chosen for transformation were held to the following criteria: (i) field experimental studies must involve mulching treatments or tillage practices, including local control treatments (conventional tillage); (ii) wheat must not be irrigated over the entire wheat growth period; (iii) for multifactorial studies, only data from conventional and mulching treatments or tillage practices must be cited, and the interactions among treatments were excluded, the five mulching practices and three tillage practices are independent factors without the interaction between them; and (iv) regarding soil water at planting, only changes in soil water at 0–3 m depth must be included. In our study, monoculture wheat was the most common cropping system; we only included studies reporting local conventional fallow (soil plowed to 16–20 cm, without other treatments until the sowing following the last wheat harvest) which always considered being a solution to the water problem facing farmers on the Loess Plateau. Additionally, the means, standard deviations (or standard errors), and sample sizes of the variables concerned must be directly available or able to be calculated from the data.

The collected data for wheat yield (kg ha^−1^) for sampling sites in Shaanxi, Gansu, Henan, Ningxia, and Shanxi on the Loess Plateau were shown in Fig. 6. In this study, WUE was defined as the ratio of grain yield and seasonal ET, with a unit of kg ha^−1^ mm^−1^; another unit of WUE widely used in the literature is kg m^−3^, which can be converted to kg ha^−1^ mm^−1^ by multiplying by 10. The field mulching practices were grouped into five types: FM, RFM, SM, MOM, and MTMC; and the tillage practices into three: NT, ST, and RT (details in Table 3).

### Data processing

The data were analyzed using the meta-analysis methods described by Hedges *et al.*^37^. The effect sizes of mulching and tillage practices for each individual observation of yield, WUE, and ET were estimated by the SMD (standardized mean difference, *g*) which was the relative value to measure the superposition between two groups, and it also reflects the differences and representative between them:
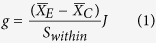

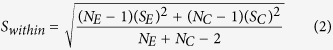

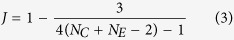

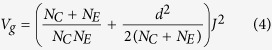
where 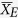 and 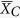 are the means of the treatment and control groups, respectively; *N*_*E*_ and *N*_*C*_ are the sample sizes for the treatment and control groups, respectively; *V*_*g*_ is the variance of independent research; S_*E*_ and *S*_*C*_ are the standard deviations for all comparisons in the treatment and control groups, respectively, and *S*_*within*_ is the comprehensive standard deviation within groups for every study. Several studies did not report standard deviations; in these cases, we calculated the average coefficient of variation (CV) within each data set and then approximated the missing standard deviations by multiplying the reported means by the average CVs.

The indexes concerned were continuous variables, and the effect sizes of different mulching and tillage practices for wheat yield, WUE, and ET were calculated by fixed or random effects models using the Review Manager program (RevMan version 5.2, 2012; Cochrane Collaboration). Inverse variance statistical methods were adopted for the meta-analysis. Random-effects models were adopted in cases of moderate to high heterogeneity (indicated by *X*^2^ >50% and a chi-square *P*-value < 0.05)^38^. The mean differences of the treatment and CT groups were weighted according to their sample sizes and SE, as determined by the RevMan program, and confidence intervals (CI) were subsequently generated from their weighted effect sizes. If the 95% CI values of the effect size for a variable did not overlap zero, the effect of a treatment on the variable was considered significant; otherwise the treatment effect was not considered significant.

### Statistical analysis

Independent-samples *t*-tests were used for pairwise comparisons of CT and alternative management practices regarding yield, ET, and WUE. Differences were evaluated at the 0.05 significance level. Pearson correlation analysis was also used to analyze the relationships between soil water at planting with grain yield, WUE, and ET for the treatments. All statistical analyses were performed using the software program SPSS, ver. 18.0.

## Additional Information

**How to cite this article**: Wang, L.- and Shangguan, Z.- Water-use efficiency of dryland wheat in response to mulching and tillage practices on the Loess Plateau. *Sci. Rep.* **5**, 12225; doi: 10.1038/srep12225 (2015).

## Supplementary Material

Supplementary Information

## Figures and Tables

**Table 1:** Meta-analysis results (heterogeneity analysis) for the effects of five mulching practices and three tillage practices on the yields, water-use efficiency (WUE), and evapotranspiration (ET) of wheat on the Loess Plateau using fixed- or random-effects models.

Items	Treatments	Categorical variable	K	P	Heterogeneity			
					Qwi	df	P-value of Chi-Square test	I^2^(%)
Yield	Mulching practices	FM	261	0.00	57.16	42	0.06	27
		RFM	166	<0.0001	33.66	28	0.21	17
		SM	132	0.26	8.67	28	1.00	0
		MTMC	103	<0.0001	7.31	19	0.99	0
		MOM	63	0.12	3.64	14	1.00	0
	Tillage practices	NT	233	0.37	29.79	32	0.58	0
		ST	139	0.05	12.58	25	0.98	0
		RT	139	<0.0001	28.08	23	0.21	18
WUE	Mulching practices	FM	208	0.01	45.69	37	0.15	19
		RTM	166	0.00	55.33	28	0.00	49
		SM	126	0.11	6.93	27	1.00	0
		MTMC	103	0.00	8.82	19	0.98	0
		MOM	63	0.12	4.42	14	0.99	0
	Tillage practices	NT	173	0.50	18.31	27	0.89	0
		ST	139	0.04	8.47	25	1.00	0
		RT	115	<0.0001	7.64	19	0.99	0
ET	Mulching practices	FM	244	0.58	27.57	39	0.91	0
		RTM	166	0.59	25.86	28	0.58	0
		SM	126	0.72	6.23	27	1.00	0
		MTMC	103	0.02	9.23	19	0.97	0
		MOM	63	0.55	1.71	14	1.00	0
	Tillage practices	NT	173	0.84	14.44	27	0.98	0
		ST	139	0.47	8.21	25	1.00	0
		RT	115	0.01	9.74	19	0.96	0

FM: flat mulching; RFM: ridge-furrow mulching; SM: straw mulching; MTMC: mulching with two materials combined; MOM: mulching with other materials; NT: no-tillage; ST: subsoiling tillage; RT: rotational tillage. K: number of observations involved in each analysis levels, including conventional tillage (CK). I^2^: heterogeneity of the individual studies; the heterogeneities of WUE with RTM could not be eliminated (chi-square P-value < 0.05).

**Table 2:** Actual values of wheat yield, evapotranspiration (ET), and water use efficiency (WUE) under various management practices on the Loess Plateau. FM, flat mulching; RFM, ridge-furrow mulching; SM, straw mulching; MTMC, mulching with two materials combined; MOM, mulching with other materials; NT, no-tillage; ST, subsoiling tillage; RT, rotational tillage; CT, conventional tillage.

Treatments	Categorical variable	*n*	Grain yield (kg ha^−1^)			WUE (kg ha^−1^mm^−1^)			ET (mm)		
			Mean	Range	Variation coefficient(%)	Mean	Range	Variation coefficient(%)	Mean	Range	Variation coefficient(%)
Mulching practices	FM	106	3851 a	259–7735	46.3	12.2 a	1.9–20.6	33.6	301 a	135–537	28.7
	CT_FM_	101	3181 b	114–6381	50.6	10.1 b	0.6–17.2	41.0	300 a	136–523	28.9
	RFM	71	3459 a	791–7898	44.6	12.4 a	5.5–26.8	33.6	290 a	126–433	27.8
	CT_RFM_	70	2831 b	255–5527	48.9	9.9 b	1.5–18.9	39.1	274 a	112–419	29.2
	SM	64	3759 a	1240–6391	36.4	11.4 a	5.7–18.0	24.3	332 a	151–503	27.7
	CT_SM_	61	3284 a	818–6033	43.0	10.0 a	3.4–15.7	32.8	329 a	148–516	26.6
	MTMC	52	4565 a	2407–6547	19.8	13.7 a	7.9–21.9	20.9	341 a	211–526	20.1
	CT_MTMC_	54	3555 b	1694–6381	27.3	11.0 b	4.9–14.5	20.3	325 a	226–523	20.0
	MOM	32	4588 a	2620–6745	21.2	13.3 a	7.2–20.1	23.5	355 a	258–555	22.1
	CT_MOM_	32	3985 b	2241–6336	25.5	11.7 b	6.0–18.1	24.1	346 a	265–541	21.8
Tillage practices	NT	87	3209 a	869–5996	39.0	10.6 a	3.5–20.1	36.7	315 a	151–523	28.9
	CT_NT_	87	3150 a	1078–5353	36.0	10.2 a	4.2–18.1	30.9	315 a	123–506	29.7
	ST	70	4270 a	1770–6909	24.0	13.1 a	7.2–23.0	23.0	333 a	204–485	20.8
	CT_ST_	70	3900 b	1548–5353	24.0	11.9 b	6.3–18.1	22.0	330 a	207–475	19.2
	RT	22	2656 a	2233–3176	11.0	10.4 a	8.3–16.8	21.9	266 a	189–314	13.4
	CT_RT_	22	2315 b	2028–2642	10.0	9.1 b	7.7–10.7	9.5	259 a	207–292	12.1

The subscripts indicate the corresponding CT for the management practices listed in the second column. Different lowercase letters in the same column indicate significant differences between single management practice and its corresponding CT (control) according to the t-test (P < 0.05).

**Table 3:** Brief descriptions of materials and methods for different mulching practices, tillage practices, and conventional tillage used in the wheat field experiments on the Loess Plateau.

Treatments	Mulching and tillage practices	Brief description
Mulching practices	Conventional tillage (CT)	The soil was plowed to 15–25 cm, without residue cover until the next sowing time (no mulching or ridge-furrowing).
	Flat mulching (FM)	The film surface was flat, and the film (7 or 8 μm thickness without color) was close to the soil surface.
	Ridge-furrow mulching (RFM)	The ridge was mulched using plastic sheets for rainwater harvesting while seeding.
	Straw mulching (SM)	Wheat straw was left unchopped or chopped and evenly distributed over the soil surface at coverage rate of 1–9 t ha^−1^.
	Mulching with other materials (MOM)	Liquid film cover, soil cover, water-permeable plastic film, or sand was used.
	Mulching with two materials combined (MTMC)	Plastic film + wheat straw, liquid film + straw, or plastic film + liquid film were used.
Tillage practices	No-tillage (NT)	The crop was planted directly into the soil with no tillage; wheat was sown with a no-tillage planter in September.
	Subsoiling tillage (ST)	In the fallow period, the soil was plowed to 30–40 cm at a certain distance interval; wheat was sown with a subsoiling tillage planter in September.
	Rotational tillage (RT)	NT was practiced for 2 years and ST for 1 year: NT/ST/NT; sub-soiling tillage in 2 years and no-tillage in 1 year: ST/NT/ST.
